# The complete mitochondrial genome of the sexual oribatid mite *Steganacarus magnus*: genome rearrangements and loss of tRNAs

**DOI:** 10.1186/1471-2164-9-532

**Published:** 2008-11-07

**Authors:** Katja Domes, Mark Maraun, Stefan Scheu, Stephen L Cameron

**Affiliations:** 1 Technische Universität Darmstadt, Institut für Zoologie, Schnittspahnstr. 3, 64287 Darmstadt, Germany; 2 Australian National Insect Collection and CSIRO Entomology, Black Mountain Laboratories, Clunies-Ross Street, Canberra, ACT 2601, Australia

## Abstract

**Background:**

Complete mitochondrial (mt) genomes and the gene rearrangements therein are increasingly used as molecular markers for investigating phylogenetic relationships, especially for elucidating deep splits. Contributing to the complete mt genomes of arthropods, especially Arachnida, available so far, we provide the first complete mt genome of a sarcoptiform mite species, the sexually reproducing oribatid mite *Steganacarus magnus *(Acari, Oribatida) which was determined by sequencing of long PCR products.

**Results:**

The mt genome of *S. magnus *lacks 16 tRNAs, only those for leucine, histidine, proline, tryptophan, glutamine and serine are present. Within those tRNAs only tRNA-His and tRNA-Pro have kept their original position, the others are translocated. Furthermore, the mt genome of *S. magnus *consists of 13,818 bp and it is composed of 13 protein-coding genes and two genes for the ribosomal RNA subunits that are typically found in metazoan mt genomes. The gene order in *S. magnus *differs from the hypothetical ancestral chelicerate arrangement as conserved in *Limulus polyphemus*: instead of *nad1-rrnL-rrnS*-LNR-*nad2 *(tRNAs excluded) *S. magnus *is *nad2-rrnL-nad1-rrnS*-LNR. Phylogenetic analyses of a concatenated amino acid dataset of all mt protein-coding genes of 28 arthropod species suggest a sister-group relationship of sarcoptiform and prostigmatid mites (*S. magnus *and *Leptotrombidium*).

**Conclusion:**

The mt gene arrangement of *S. magnus *differs from the hypothetical ground plan of arthropods and from that of other mites further contributing to the variety of mt gene arrangements found in Arachnida. The unexpected lack of tRNAs is enigmatic, probably showing that the loss of mt genes is an ongoing evolutionary process. For solving phylogenetic relationships of oribatid mite lineages and their position within Acari further complete mt genomes are needed.

## Background

Mitochondria are maternally inherited cell organelles that contain a circular genome of about 14–19 kb in bilaterian animals; the mitochondrial (mt) DNA in metazoans usually codes for 13 proteins, 22 transfer RNAs (tRNA), two ribosomal RNAs (rRNA; large (*rrnL*) and small (*rrnS*) ribosomal subunit) and contains a non-coding control region (LNR) of variable length [1,2]. The loss of genes in mitochondria is a commonly recognized and ongoing process in eukaryotes [3]. Eukaryotic mt genomes generally contain fewer genes than their free-living bacterial ancestors since the majority of the original mt proteins are now encoded in the nucleus; this is either caused by the transfer of the original mt gene to the nucleus or by the replacement of its function by a preexisting nuclear gene [4]. The protein-coding genes which have been retained in mt genomes are mainly those involved in electron transport and phosphorylation, e.g., cytochrome b (*cob*) and the cytochrome oxidases (*cox1, cox2, cox3*) [4], but their number is variable ranging from three in the malaria parasite *Plasmodium falsciparum *(Apicomplexa) [5] to 67 in *Reclinomonas americana*, the earliest branching aerobic protist (Chlorophyta) [6].

In addition to gene loss, the positions of genes relative to each other exhibit frequent rearrangement. While the arrangement of mt genes is conserved in some lineages of arthropods [7,8], it is highly variable in others [9-11]. In particular, the positions of the relatively small genes for tRNAs frequently vary within and among taxa. The arrangement of the hypothetical ancestor of arthropods is conserved in the horseshoe crab *Limulus polyphemus *[12], whereas most insect genomes differ from the ancestral state by the location of one tRNA [7].

Oribatid mites (Acari, Oribatida) are soil-dwelling animals that occur in high numbers in almost all terrestrial ecosystems [13]. A characteristic feature of this group is the unusually high percentage of parthenogenetic taxa (~10% of all species) and the co-occurrence of sexuality and parthenogenesis in the same habitat [14]. Oribatid mite fossils date back at least 360 million years [15,16] and therefore a number of parthenogenetic lineages of oribatid mites join bdelloid rotifers as "ancient asexual scandals" [17-20]. Since oribatid mites provide insights into the evolution and maintenance of sex, recently much attention has been paid to their phylogeny and radiation [21-24]. However, studies based on single genes such as the ribosomal 18S region (18S), the heat shock protein 82 (*hsp82*), the elongation factor 1α (*ef1α*) or *cox1 *could neither satisfactorily resolve phylogenetic relationships [25] nor clarify the number of parthenogenetic radiations [21] nor delineate the age of the group [20]. Since oribatid mites apparently are among the first terrestrial animals (I. Schaefer, R.A. Norton, S. Scheu & M. Maraun, unpubl. data) and species exhibit different evolutionary mutation rates, lineages are probably vulnerable to long-branch attraction in phylogenetic reconstruction. Therefore, further markers, such as gene rearrangements in mt genomes, are needed to resolve phylogenetic relationships of oribatid mite lineages, among mite taxa (Acari) and among chelicerates in general.

Until now no complete mt genome of an oribatid mite species was available although mitochondrial genomes have become invaluable phylogenetic markers during the last few years. Complete mt genome sequences are now known for about 150 arthropods, including 26 chelicerates with 15 species of Acari. These 15 acarine genomes represent ten species of ticks (Ixodida, Parasitiformes), two mesostigmate mites (Mesostigmata, Parasitiformes) and three species of the genus *Leptotrombidium *(Prostigmata, Acariformes). In contrast to other arthropods and Metazoa in general [2], the arrangement of the mt genes differs markedly within and among taxa of Acari. While the ancestral state of arthropods is retained in soft- and prostriate-hard ticks [8,26], there is a major rearrangement shared by all metastriate hard ticks [26,27], and there are numerous, lineage specific rearrangements in the mesostigmate species, such as *Varroa destructor *[28] and *Metaseiulus occidentalis *[29], and also in the prostigmate mite genus *Leptotrombidium *[10]. Since the deep phylogeny of Acari, especially within the large subgroup of Acariformes, is still controversial, synapomorphic rearrangements of mt genes, if present, would likely allow new insights into phylogenetic relationships.

We report the first mt genome for an oribatid mite, *Steganacarus magnus *(Nicolet, 1855). The mt genome of *S. magnus *is compared with that of other mites and with basal chelicerates to clarify phylogenetic relationships within Acari and Chelicerata in total. We show that the mt genome of *S. magnus *is slightly rearranged and lacks 16 tRNA genes.

## Results and discussion

### Mitochondrial genome organization

**Figure 1:**
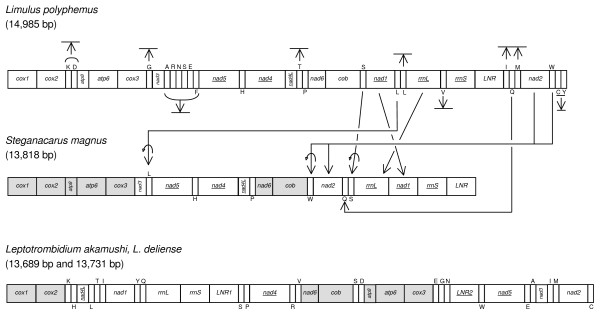
**Mitochondrial genome organization of *Limulus polyphemus*, *Steganacarus magnus *and two *Leptotrombidium *species**. Arrows indicate gene rearrangements (translocations, inversions and loss). Underlined genes and tRNAs with letters below are encoded in the opposite strand. Shaded boxes indicate shared gene boundaries between *S. magnus *and *Leptotrombidium*. Gene abbreviations: *cox1–3*: cytochrome oxidase subunits 1–3; *atp6,8*: ATP synthase subunit 6,8; *nad1–6, 4L*: NADH dehydrogenase subunit 1–6, 4L; *cob*: cytochrome b; *rrnL*: large ribosomal subunit; *rrnS*: small ribosomal subunit; LNR: large non-coding control region. tRNA genes are symbolised by the one-letter code of their amino acid: I = Isoleucine; Q = Glutamine; M = Methione; W = Tryptophane; C = Cysteine; Y = Tyrosine; K = Lysine; D = Aspartate; G = Glycine; A = Alanine; R = Arginine; N = Asparagine; S = Serine; E = Glutamate; F = Phenylalanine; H = Histidine; T = Threonine; P = Proline; L = Leucine; V = Valine.

The mt genome of *S. magnus *is the first genome published for the large mite group of Sarcoptiformes. The complete mt genome is circular and consists of 13,818 bp [GenBank: EU935607]. It encodes 13 protein-coding genes, two rRNA genes, six tRNA genes and includes a large non-coding control region as well as several size-variable intergene spacer regions (Fig. 1, Additional File 1). Genes are encoded on both strands which is typical for arthropods. Compared to the mt arrangement of the ancestral arthropod, which is conserved in *Limulus polyphemus *[12], the genome of *S. magnus *is slightly rearranged: instead of *nad1-rrnL-rrnS*-LNR-*nad2 *(tRNAs excluded, underlined genes are coded on the minority strand) the gene arrangement is *nad2-rrnL-nad1-rrnS*-LNR (Fig. 1); all translocated genes have kept their original orientation. Furthermore, the genes for tRNA-Leu, -Trp, -Gln and -Ser are translocated to new positions with tRNA-Ser, -Leu, and -Trp also being inverted relative to the ground plan; only tRNA-His and -Pro remained in their original location.

Rearrangements of genes in mt genomes are useful markers for deep splits within phylogenies [2] although the relative frequency of rearrangements varies among lineages [30]. For example, *Drosophila *(Insecta) and *Daphnia *(Crustacea) share the same mt rearrangement although they diverged 400–500 million years ago [30] and the mt genome of soft ticks remained unchanged for a similar time period [8]. In contrast, gene arrangements in mt genomes of lice are highly variable [9,11], and in the mite genus *Leptotrombidium *they even differ between species [10]. In general, in mt genomes of Acari a number of rearrangements have occurred. While the ground plan is retained in soft- and prostriate-hard ticks [26], the arrangement of mt genes in Mesostigmata (Parasitiformes), especially *Metaseiulus occidentalis *is strongly derived. Notably, the *Metaseiulus *mt genome is the largest within chelicerates, even though *nad3 *and *nad6 *were lost, due to the duplication of many of the remaining genes [29].

Fossils of oribatid mites are known from Devonian sediments and molecular studies suggest that their origin may predate this record by ~180 mya (I. Schaefer, R.A. Norton, S. Scheu & M. Maraun, unpubl. data). Further, oribatids probably diverged from other acariform mites, such as *Leptotrombidium*, about 570 mya ago (I. Schaefer, R.A. Norton, S. Scheu & M. Maraun, unpubl. data). The mt genomes of *Leptotrombidium *and *S. magnus *differ markedly; they only share the gene boundaries *cox1-cox2*, *nad6-cob *and *atp8-atp6-cox3 *(Fig. 1). However, since these boundaries are also present in the ground plan of chelicerate mt genomes there are no derived arrangements supporting the common ancestry of Acariformes.

### Protein-coding genes and nucleotide composition

All 13 protein-coding genes typically present in arthropods could be identified in the mt genome of *S. magnus*. They start with the mt start codons ATT, ATG, ATA and ATC (Additional File 1). Six genes (*cox1*, *atp6*, *nad3*, *nad5*, *cob*, *nad2*) terminate with incomplete stop codons (T or TA; Additional File 1) while all others terminate with either TAA, TAG or TTT (*atp8*, *cox3*, *nad4*, *nad4L*, *nad6*, *nad1*; Additional File 1). In *cox2*, which is flanked by two other protein-coding genes (*cox1 *and *atp8*), no stop codon is present; as shown for sets of protein-coding genes in *Anabrus simplex *(Orthoptera, Insecta) a stem-loop formation in the secondary structure of the transcribed polycistronic mRNA probably functions as terminator [31].

The percentage nucleotide composition of the mt (+)-strand is A = 36.5, C = 13.2, G = 12.2 and T = 38.1. Therefore, there are approximately equal numbers of each complementary nucleotide pairs (A:T, G:C) but a strong AT-bias is present. The pattern for all protein-coding genes is also strongly AT-biased but with a much higher T than A content (Additional File 1). Skews calculated for neutral fourfold degenerate sites do not indicate consistent asymmetric strand bias (Additional File 1). Genes encoded on the (+)-strand show either neutral (*atp6, nad2*), positive (*cox2*, *nad6*) or negative CG-skew (*cox1*, *cox3*, *nad3*, *cob*). The majority of genes encoded on the (-)-strand are positive CG-skewed (*nad5*, *nad4L*, *nad1*) (Additional File 1). The AT skew at fourfold degenerate sites is only positive for *nad2 *and *nad5*, but negative for all other protein-coding genes (Additional File 1).

A reversal of the strand bias is usually explained by an inversion of the control region (LNR) which contains the origin of replication and translation [2,32]. During replication the two different strands ((-)- and (+)-strand) are exposed to different mutational pressures, typically causing distinct skews since one strand remains longer in the single-stranded state than the other [33]. Therefore, the LNR likely functions as a key region for determining strand bias and an inversion results in a complete reversal of the strand nucleotide composition over time [33]. In *S. magnus *most genes encoded on the (+)-strand show a negative CG-skew at fourfold degenerate third codon positions which is inverted to the common pattern and probably indicates a reversal of the LNR. On the other hand, the presence of neutral or positive skewed genes may indicate that this reversal is of recent origin and consequently the process of inverting nucleotide skew is not completed so far.

The absence of a distinct strand bias can also be explained by the recent inversion of single genes which homogenize general patterns of asymmetry [33,34]. However, since no inversions of protein-coding genes were found in *S. magnus*, the absence of a distinct asymmetrical skew in the genome awaits explanation.

### Putative control region

The major non-coding region (LNR), which presumably functions as the mitochondrial control region, is 1019 bp in length and located between *rrnS *and *cox1 *(Fig. 1). There are additional non-coding intergenic regions ranging in size from 2–127 bp. These regions were blasted and checked for tRNA genes but could not be assigned to any functional gene.

**Figure 2:**
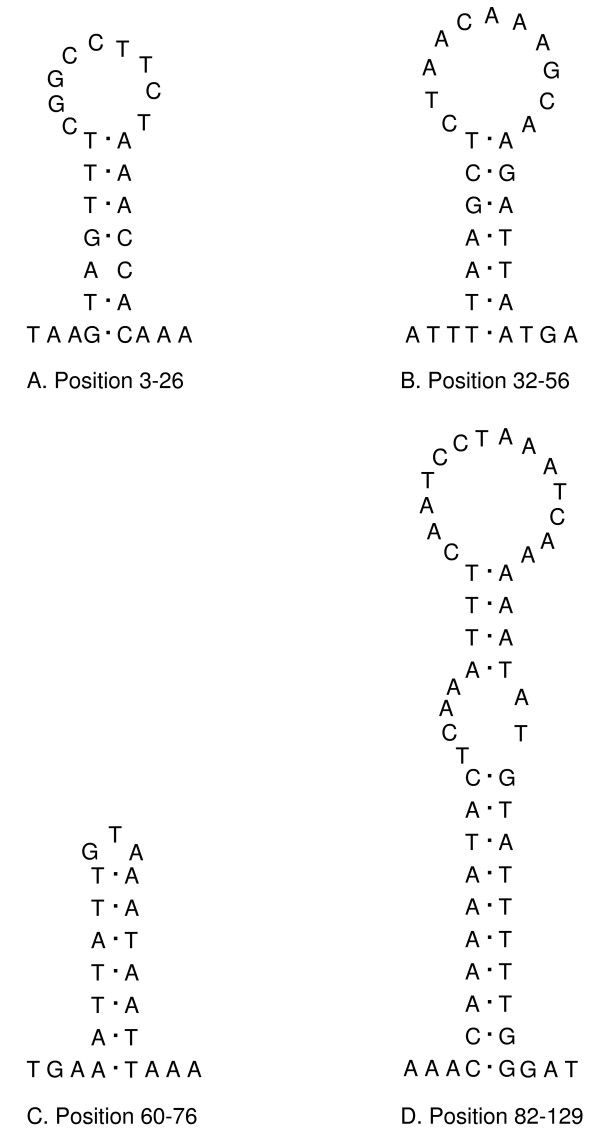
**Putative control region secondary structures**. Possible stem-loop structures of the origin of replication in the putative control region within the mitochondrial genome of *Steganacarus magnus*.

The relative location of the LNR varies greatly among invertebrates with the ancestral pattern of arthropods being *rrnS*-LNR-tRNA-Ile [1,2]. It also varies in length mostly due to different numbers of sequence repeats, and length heteroplasmy within individuals has also been recorded [32]. There was no length heteroplasmy in *S. magnus *but two inverted sequence repeat regions, each with a length of 190 bp, were present at positions 133–322 (repeat 1) and 830–1019 (repeat 2). The region before repeat 1 contains four stem-loop structures at positions 3–26, 32–56, 60–76 and 82–129 (Fig. 2); the region between the two repeats contains 10–12 stem-loop structures depending on differences in folding. None of the hairpin structures is associated with a poly-A or poly-T stretch which would mark the origin of replication (OR) in insect mt genomes [32] nor with a TATA- or GA(A)T-motif as present in other arthropods [26]. Since the OR typically is close to the gene of *rrnS *and repeat regions are posterior to it [32], we assume the first region to be the OR of the mt genome of *S. magnus*.

In the mesostigmate mite *M. occidentalis *the stem-loop structure which probably represents the OR comprises only AT nucleotides but does not have any similarity to sequences from other chelicerates [29]. The LNR of the mite *V. destructor *includes several repetitions of a 157-bp motif and eleven sites of potential stem-loop structures have been identified close to it [28]. In the genus *Leptotrombidium*, the closest relative to *S. magnus *for which an mt genome is sequenced, the LNR is duplicated (and one is inverted) in *L. akamushi *and *L. deliense *and four copies are present in *L. pallidum *[10,27]. Although mt LNR possess several distinct structural features (e.g., high AT-content, concerted evolution of tandem repeats, stem-loop structures), their use for evolutionary studies is limited by the high variability of the sequence and the possibility of length heteroplasmy within individuals [32].

### rRNA genes

**Figure 3:**
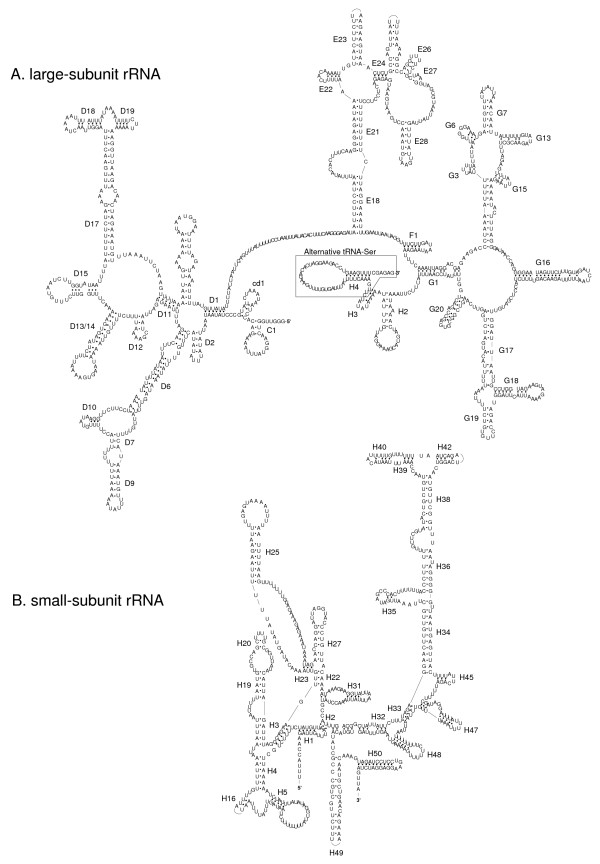
**Putative secondary structure of the large- (A) and small-subunit ribosomal RNA (B) of *Steganacarus magnus***. Dots indicate complementary nucleotide bonds.

The large subunit of the rRNA (*rrnL*) is 992 bp in length (Additional File 1) which is a bit shorter than in other mite species (e.g., about 1,014 bp in *Leptotrombidium *[10], 1,212 bp in *Carios capensis *[8]). The 5'-end starts three nucleotides apart from *nad1 *(encoded on the (-)-strand). The 3'-end was difficult to assign since parts of the last stem-loop structure can be included in the gene for tRNA-Ser (Fig. 3A). The gene for the small subunit (*rrnS*) is 609 bp in length and located between *nad1 *and the control region (Additional File 1, Fig. 3B). Both ribosomal subunits have a similar AT content to the protein-coding genes and both are encoded on the (-)-strand as in most species of arthropods and chelicerates (e.g., *Limulus*, Araneae, Ricinulei, Pycnogonida, Scorpiones and Ixodidae) [12,26,35-37]. In contrast, in the mt genome of the closely related genus *Leptotrombidum *as well as in *M. occidentalis *both ribosomal RNA genes are encoded on the (+)-strand (but *L. pallidum *has a duplicated *rrnL *gene on the (-)-strand) [10,27,29].

The secondary structures of the rRNA genes in *Steganacarus *differ from those published for *Leptotrombidium *[10] but are more similar to those published for insects [e.g., [38,39]]. The *Leptotrombium rrnS *gene lacks helices 1, 2, 4, 5, 7, 8 and 22, and the compound helices 19–20–21 and 39–40–42 as depicted have a very different secondary structure from that found in other arthropods [10]. In contrast, most of the helices found in arthropod *rrnS *genes are present in *Steganacarus*, but helices 7 and 8 are absent. However, helix 5 has a large loop such that there is limited difference in sequence length in this region between *Steganacarus *and arthropods which possess these helices; this is similar to the structure found in Hymenoptera [38]. Helix 16 is greatly shortened relative to other species, consisting of just 2 paired bases compared to up to 8 in *Leptotrombidium *[10]. The loop regions between helices 38 and 39–40–42 are also greatly reduced consisting of just 4 bp on the 5' side and 2 bp on the 3'; this is in contrast to insects where these loops consist of a dozen or more bases on each side.

Similarly, the *rrnL *gene of *Leptotrombidium *entirely lacks domain I (helices B12, B20), helices C1, cd1, D1 and H3 and the structure of the compound helices D17-D18-D19 is unique to *Leptotrombidium*. The *Steganacarus rrnL *secondary structure is again more similar to that of other arthropods, domain I is absent but helices C1, cd1 and D1 are present and D17-D18-D19 has a more canonical structure. Helix G3 is greatly reduced, consisting of just 2 paired stem bases and 3 loop bases, relative to both *Leptotrombidium*, 6 stem and 3 loop bases, and insects, up to 20 stem and 22 loop bases in *Manduca *[39]. The 3' end of the *rrnL *molecule is ambiguous, bases 10346–10290 either form the tRNA gene for Serine or they form helices H3 and the 3' side of H2. Without an analysis of the mature transcribed genes it is not possible to determine which form is more likely in the mature molecule. Helix H3 is not present in all arthropod *rrnL *genes found in Hymenoptera and Lepidoptera but is absent from Coleoptera and *Leptotrombidium *and its function in the mature rRNA is unclear. Accordingly, we present both possibilities, helices H2 and H3 are included in Figure 3 with the region which potentially forms the tRNA shown in a box, while tRNA-Ser is included in Figure 1 and Additional File 1.

### tRNA genes

Out of the 22 tRNA genes typically present in arthopods only six are present in *S. magnus*. Out of these six tRNAs, only two have kept their original position (tRNA-His between *nad5 *and *nad4*, tRNA-Pro between *nad4L *and *nad6 *with tRNA-Thr missing) while all others are translocated relative to the ground plan. Further, tRNA-Leu overlaps the *nad5 *gene by four nucleotides and tRNA-Trp overlaps with the 3'-end of *cob *by 16 nucleotides (Additional File 1); as described above tRNA-Ser presumably forms part of the *rrnL *gene at the 3'-end (Fig. 3A). Remarkably, although 16 tRNA genes have been lost, the mt genome size of *S. magnus *(13,818) is comparable to those of *Leptotrombidium deliense *(13,731) and *L. akamushi *(13,698) [10,27] which is due to a larger LNR and more intergenic spacer regions.

**Figure 4:**
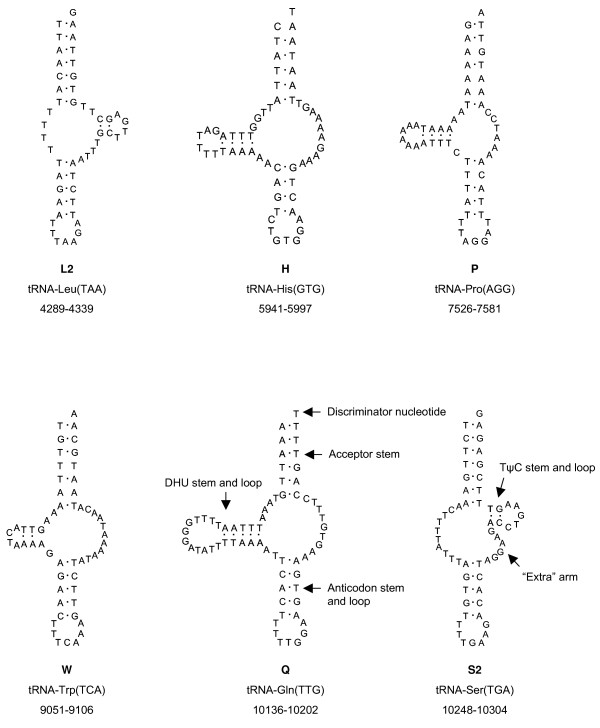
**tRNA secondary structures of *Steganacarus magnus***. tRNAs are labelled with the abbreviations of their corresponding amino acids.

All present tRNAs differ remarkably from the typical cloverleaf structure: in tRNA-Leu and -Ser the D-stem and -loop are missing and the TψC-stem is short with only two complementary base pairs (Fig. 4). In contrast, the homologous tRNA for leucine in *L. pallidum *and *M. occidentalis *lacks the TψC- instead of the D-arm [27,29]; while the structure of tRNA-Ser in *L. pallidum *is similar to that of *S. magnus*, it differs in *M. occidentalis *which lacks the TψC-arm.

The tRNAs for histidine, proline, tryptophan and glutamine lack the TψC-stem and -loop but posses a complete DHU-stem with a smaller loop in tRNA-Trp and a larger in tRNA-Gln (Fig. 4); this lack of the TψC-arm is also present in *L. pallidum *[27] and *M. occidentalis *[29].

All tRNAs present in *S. magnus *are shorter than the average length of arthropod tRNAs (about 66 bp) and are highly modified. The loss of the TψC-arm and the replacement by a size-variable loop was first recognized in nematode tRNAs [40] and can also be found in *M. occidentalis *[29], *L. pallidum *[10] and other chelicerates including scorpions [35] and spiders [41]. Further, a study on truncated tRNAs in Arachnida revealed that the tRNAs for proline, histidine and glutamine have experienced the greatest number of independent TψC-arm losses in arachnids whereas TψC-arm loss in genes for arginine, lysine and methione have occurred only once and is synapomorphic for opisthothele spiders [42]. However, arachnids seem to have a compensatory mechanism that allows truncated tRNAs to function during translation and the interaction with the ribosome [42].

While the two rRNA genes are present in all eukaryotic genomes [4], the number of tRNA genes varies markedly among taxa. No tRNAs are present in the protists *Plasmodium falciparum *(Apicomplexa) and *Trypanosoma brucei *(Kinetoplastida) [43] but up to 27 are present in *Reclinomonas americana *(Chlorophyta) [6]. The loss of mt tRNA genes may be facilitated by the fact that the proteins with which they interact during translation in the mitochondria are encoded in the nucleus [44]. Furthermore, the mutation rate of mt encoded tRNA genes is about five-fold higher than that of nuclear genes which experience strong purifying selection [45,46]. Since tRNAs are encoded in the nucleus anyway and selective pressure favors the reduction of mt genome size [4], it is likely that *S. magnus *simply lost its tRNA genes instead of transferring them to the nucleus. However, the differences in the genetic code between nucleus (universal code) and mitochondria (invertebrate mt code) would probably argue for two sets of tRNAs or alternatively some modifications of the tRNA-amino acyl transferases are needed to treat each tRNA isotype differently in the two different compartments.

### Phylogenetic analysis

**Figure 5:**
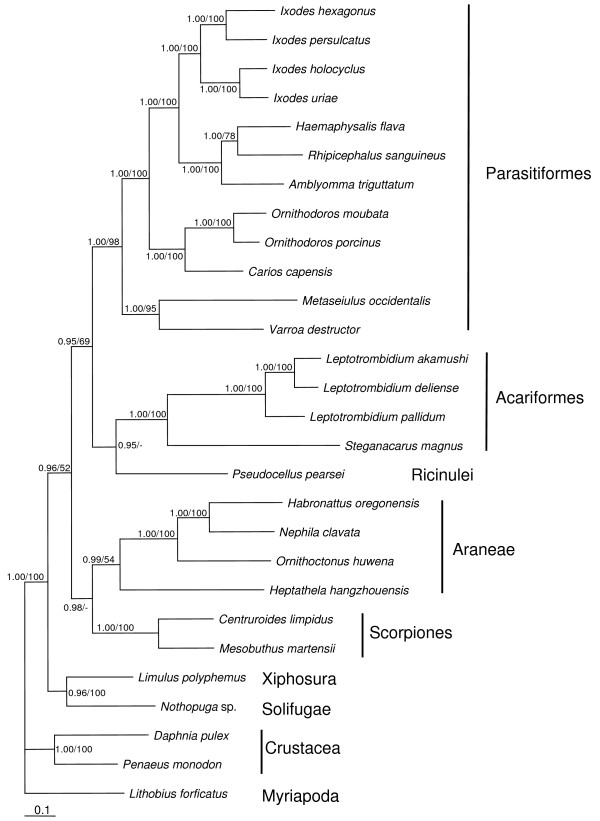
**Bayesian tree phylogeny**. Bayesian tree of a concatenated amino acid dataset of all mitochondrial protein-coding genes in the order *atp8, atp6, cox1, cox2, cox3, cob, nad1, nad2, nad3, nad4, nad4L, nad5 *and *nad6 *of 28 arthropod species. Myriapoda are used as outgroup. Numbers at nodes represent posterior probabilities for Bayesian analyses and bootstrap support values for neighbor joining.

Phylogenetic analyses performed on a concatenated dataset of all protein-coding genes (amino acid sequences) included three outgroup species (*Daphnia pulex *(Crustacea), *Penaeus monodon *(Crustacea), *Lithobius forficatus *(Myriapoda)), one species of Solifugae, Xiphosura, and Ricinulei, two Scorpiones species, four species of Araneae and Acariformes and twelve species of Parasitiformes (Fig. 5). All major groups, notably Parasitiformes, Acariformes, Araneae and Scorpiones, were monophyletic and supported by moderate (Araneae) to high (Acariformes, Scorpiones) support values. The sister-group relationship of Scorpiones and Araneae was only supported by posterior probablities (Fig. 5).

The newly sequenced *S. magnus *formed the closest relative of the prostigmate mite genus *Leptotrombidium *which was expected following previous studies [13]. As in previous studies [36] the ricinuleid species *Pseudocellus pearsei *clustered as sister-group of Acariformes but was only supported by Bayesian posterior probabilities (Fig. 5). In contrast to the study of Fahrein et al. [36] the Acariformes/Ricinulei clade did not form the sister-group of Araneae but of Parasitiformes; however, the support for both possibilities is similar weak [see [36]].

For a broader investigation of chelicerate phylogeny mt DNA data of key taxa such as Opiliones, Pseudoscorpiones, Palpigradi, Uropygi and Amblypygi are missing. Even for a complete study of Acari phylogeny many taxa remain to be sampled; no complete mt genome is available for Astigmata, Endeostigmata or Opilioacarida.

While using mt DNA for phylogenetic studies, a reversal of nucleotide strand bias or a reversal of nucleotide bias of single genes (caused by gene inversion or the inversion of the control region) can be misleading for phylogenetic relationships by causing long-branch attraction artifacts [34]. Further, since mt genomes evolve at higher rates than the nuclear genome [47], saturation of the phylogenetic signal can also be problematic in deep split phylogenies. Species exhibiting unusual genomic features such as complicated gene arrangements or multiple control regions as well as species with small body sizes or parasitic lifestyle are also vulnerable to long branches [34]. Collectively, Acari exhibit all these features and so a heavy sampling effort will be necessary to reliably use mt genomics in mite phylogenetic studies.

## Conclusion

The first complete mt genome for an oribatid mite (*S. magnus*, Oribatida, Acariformes) is a typical circular molecule and comprises all protein-coding genes typically present in Metazoa and two genes for the ribosomal subunits. Compared to the putative ground pattern of arthropods the genome is rearranged, affecting the genes *nad1, rrnL, rrnS*, and *nad3*. Further, the genome of *S. magnus *lacks all but six tRNAs, but is comparable in size with genomes of the closely related genus *Leptotrobidium*; the close relationship of both of these acariform mites was confirmed by phylogenetic analyses using all mt protein-coding genes. Since mt gene arrangements vary strongly among mite species and no full mt genomes are available for Astigmata, Endeostigmata and Opilioacarida, the use of mtDNA rearrangements for phylogenetic studies is limited at present, especially since gene rearrangements in the studied Acari species do not show a distinct phylogenetic pattern. However, the growing number of published genomes and the better understanding of rearranging mechanisms make mt genomes promising markers for resolving phylogenetic relationships of acarine lineages and Chelicerata in general.

## Methods

### DNA processing

Specimens of *S. magnus *were collected from the Kranichstein forest located about 8 km northeast of Darmstadt, Germany. Animals were extracted from leaf litter by heat using a modified Kempson extractor [48], preserved in 75% ethanol and stored at -20°C until usage. Total DNA was extracted from single specimens using the DNeasy Tissue kit (Qiagen) following the manufactures' protocol (but final elution of DNA was in 40 μl instead of 200 μl). Polymerase chain reactions (PCR) were performed for the small ribosomal subunit (*rrnS*), the cytochrome b (*cob*) and cytochrome oxidase I (*cox1*) genes using the primers 12SA and 12SB, CB3 and CB4 and COIarch1 and COIarch2, respectively (Additional File 2). PCR reaction mixtures contained 12.5 μl HotStarTaq MasterMix (Qiagen), 0.7 μl of each primer (100 pmol/μl), and 4 μl DNA (unquantified) in a total volume of 25 μl. Amplification conditions included an initial activation step at 95°C for 15 min followed by 34 cycles of 95°C for 45 s; 44°C (*rrnS*), 50°C (*cob*) or 51°C (*cox1*) for 1 min; 72°C for 55 s and a final elongation at 72°C for 10 min. PCR products were visualized on a 1% agarose gel, purified using the QIAquickPCR Purification kit (Qiagen) and directly sequenced by Macrogen (South Korea).

Long PCR amplifications were performed using 1 μl Elongase (Invitrogen), 1 μl buffer A, 4 μl buffer B, 2.5 μl dNTPs (25 mM), 1 μl of each primer (10 mM) and 1–2 μl of DNA (unquantified) with the following conditions: 92°C for 2 min; 40 cycles of 92°C for 30 s, 50°C for 30 s, 68°C for 12 min and a final extension step of 68°C for 20 min. Initial primers for long PCR were designed from the previously obtained sequences of *rrnS*, *cob *and *cox1 *(Additional File 2). PCR products were visualized on 1% agarose gels and purified using Millipore Montage vacuum purification plates. Sequencing was performed using ABI BigDye 3.1 dye terminator technology with an ABI capillary sequencer at the John Curtin Medical School sequencing centre (Australian National University). Cycle sequencing reactions contained 1 μl BigDye, 0.5 μl of the primer (25 mM) and 0.5–1.0 μl template (in a total volume of 3 μl) and amplification conditions were 28 cycles of 94°C for 10 s, 50°C for 5 s and 60°C for 4 min. Within each long PCR product the complete double-stranded sequence was determined by primer walking (a list of all primers is available from the corresponding author upon request). Since PCR amplifications derived from DNA extracted from different specimens the final genome sequence is a consensus from a pool of individuals.

### Analysis and annotation

Data were assembled into contigs using Sequencher™ version 4.7 (Gene Codes Corperation 2006). Protein-coding genes (PCG) were identified by the comparison of their amino-acid sequences using the blastx search BLAST algorithm implemented at the NCBI website  and by eye-comparison with other chelicerate sequences. Annotation of the N- and C-terminal ends of each PCG was checked by comparison with the translated amino acid sequences of homologous mt genes for other chelicerates in MEGA ver. 3.1 [49]; MEGA ver. 3.1 was also used for nucleotide composition analyses.

tRNA genes were initially identified by tRNAScan-SE [50] using both the generalized mitochondrial and the specific nematode mitochondrial tRNA settings and by the program ARWEN [51]. Genes found were adjusted by eye to identify structures more similar to those found in other chelicerates [cf. [42]]. Non-coding regions were also searched by eye for stem-loop motifs which could form part of plausible tRNA-like structures but none were found. While it is possible that additional tRNAs are present but unannotated in the *Steganacarus *mt genome they cannot be identified at this time.

Candidate rRNA genes were identified by blastn searches and aligned with homologous rRNA genes from other chelicerates and insects. A secondary structure model for each gene was elucidated by comparison to the published rRNA secondary structures for *Apis *[38], *Manduca *[39] and *Leptotrombidium *[10].

For phylogenetic analyses we used the concatenated amino acid dataset of all protein-coding genes previously used by Fahrein et al. [36]; amino acid sequences were choosen since a more conservative and more unambiguous alignment was possible. Sequences were aligned using the default settings in ClustalX 1.81 [52]; the gene order was adapted from Fahrein et al. [36]. Since most parts of the aligned amino acid sequences were unambiguous all parts were included in tree construction. Bayesian phylogenetic analysis was performed with MrBayes v3.1.2 [53] using the mitochondrial genetic code for Metazoa and a haploid ploidy level with two independent runs of 1,000,000 generations and four chains each; trees were sampled every 500 generations. A majority consensus tree was generated using a burn-in of 200. Phylogenetic trees were also constructed using neighbour-joining (NJ) based on uncorrected p-distances as implemented in PAUP* 4b10 [54]. Reliability of the branches was ascertained by bootstrap analyses with 10,000 replicates.

## Authors' contributions

KD carried out the molecular studies, performed the phylogenetic analysis and drafted the manuscript. SLC participated in the design of the study, supervised the lab work and the genome annotation and did the secondary structure analyses; he also co-wrote the manuscript. SS conceived of the study, participated in its design and coordination and helped to draft the manuscript. MM also participated in the design of the study and helped to draft the manuscript. All authors read and approved the final manuscript.

## Supplementary Material

Additional File 1 **Annotation of the mitochondrial genome of *Steganacarus magnus***. ^1^AT skew = (A-T)/(A+T), ^2^GC skew = (C-G)/(C+G), *skews at 4-fold degenerate sites. Click here for file

Additional File 2 **Initial primers for PCR amplifications of the mitochondrial genome of *Steganacarus magnus***. *rrnS *= small ribosomal subunit, *cob *= cytochrome b, *cox1 *= cytochrome c oxidase subunit I, * used with Steg 5. Click here for file
